# A model for the spread of infectious diseases compatible with case data

**DOI:** 10.1098/rspa.2021.0551

**Published:** 2021-10

**Authors:** Norden E. Huang, Fangli Qiao, Qian Wang, Hong Qian, Ka-Kit Tung

**Affiliations:** ^1^ Data Analysis Laboratory, First Institute of Oceanography, Qingdao 266061, People's Republic of China; ^2^ Shanghai Jiao Tong University, Shanghai 200240, People's Republic of China; ^3^ Department of Applied Mathematics, University of Washington, Seattle, WA 98195, USA

**Keywords:** SIR and SEIR models, COVID-19, herd immunity, basic reproduction number, vaccination

## Abstract

For epidemics such as COVID-19, with a significant population having asymptomatic, untested infection, model predictions are often not compatible with data reported only for the cases confirmed by laboratory tests. Additionally, most compartmental models have instantaneous recovery from infection, contrary to observation. Tuning such models with observed data to obtain the unknown infection rate is an ill-posed problem. Here, we derive from the first principle an epidemiological model with delay between the newly infected (*N*) and recovered (*R*) populations. To overcome the challenge of incompatibility between model and case data, we solve for the ratios of the observed quantities and show that log(*N*(*t*)/*R*(*t*)) should follow a straight line. This simple prediction tool is accurate in hindcasts verified using data for China and Italy. In traditional epidemiology, an epidemic wanes when much of the population is infected so that ‘herd immunity’ is achieved. For a highly contagious and deadly disease, herd immunity is not a feasible goal without human intervention or vaccines. Even before the availability of vaccines, the epidemic was suppressed with social measures in China and South Korea with much less than 5% of the population infected. Effects of social behaviour should be and are incorporated in our model.

## 1. Introduction

Almost 100 years ago, a classic paper published in *Proceedings of the Royal Society* by Kermack & McKendrick [1], entitled ‘A contribution to the mathematical theory of epidemics’, started the tradition of mathematical modelling on the spread of infectious disease among a susceptible population. Current thinking in epidemiology is deeply rooted in concepts introduced in that paper, some of which are still relevant, while others need to be modified. The simple mathematical model they introduced is called the SIR model, for susceptible–infected–removed:

1.1 $$\frac{\text{d}}{\text{d}t}I=aSI-bI;\;\frac{\text{d}}{\text{d}t}S=-aSI;\;\frac{\text{d}}{\text{d}t}\tilde{R}=bI.$$

When a few infected individuals are introduced into a susceptible population *S*(*t*), it leads to the growth of the active infected population *I*(*t*). The infected individuals eventually recovered, becoming $\tilde{R}(t)$ and in the process acquiring immunity to the original infectious disease, or dead and no longer infectious. The recovered and the death are lumped together as the ‘removed’: $\tilde{R}(t)$. The removed population in the SIR model is an accumulated population, denoted here as $\tilde{R}(t)$, to distinguish it from the rate of removal that we will use in this paper, which will be denoted by *R*(*t*). An epidemic wanes in the SIR framework when the susceptible fraction of the population is gradually depleted, achieving ‘herd immunity’, so that the number of susceptible persons an infected individual can infect, as measured by the reproduction number, falls to below one.

There have been many variants to this basic model. One common modification is to add an extra population of *E*(*t*) for exposed individuals who are not yet infectious, in the so-called SEIR model (e.g. [2]). Other—agent-based—models take advantage of the modern computing power to further subdivide the population into many subgroups and even simulate movements of individuals (e.g. [3]). But basic concepts are similar to the original SIR model. These mechanistic models play an important role before the outbreak spreads, since the models could be used to explore various scenarios for policy decisions on social distancing and lockdown. By constantly updating an SEIR model with real-time statistics on transmission from mobility and serological data, and death rates among different age groups, real-time monitoring of the epidemic can also be provided by the model and the effect of lockdown evaluated for England during the first wave [4]. Another data-driven SEIR model with updates on hospital admission data and mobility data, plus some imposed percentage of asymptomatically infected, was used to inform policy-makers on the effects of lockdown on COVID-19 in Ile-de-France, and on exit strategies [5]. Discussion on these types of models used in modelling the current COVID-19 epidemic and their roles can be found in [6].

Two challenges facing modellers are: first, there is an incompatibility between observational data and model output: for COVID-19, a large fraction of the infected population have no or mild symptoms, but are nevertheless infectious. Only those who are more seriously sick are admitted to hospitals and/or tested for COVID-19. Model outputs are for the total infectious population, whether tested or not and whether asymptomatic or not. The data, on the other hand, are for ‘confirmed cases’, after the subset of the infected is tested. Second, compartmental models, such as SIR and SEIR, assume instantaneous recovery from infection. The statistical justification is based on exponential waiting time for the first event to occur (in this case, the first person to recover among all infected people), and this waiting time approaches zero as the population size approaches infinity [7]. This is more applicable to radioactive decay of a large ensemble of atoms than to the biological process of recovery in a small population of infected. At least for the current COVID-19 pandemic, this is contrary to what our data show. These two factors make it inappropriate to use these models in a data-driven way, because they are inconsistent with data. We will provide a reformulation of the epidemic model to address these two challenges.

There can actually be two possible end states of an epidemic: one is through ‘herd immunity’ mentioned above, and the other is an unstable state achieved by suppressing contacts and hence transmission among individuals, called ‘suppressed equilibrium’ here. This second state is unstable (parametrically) because, if the social distancing measures are relaxed and the businesses and schools reopened, the disease could initiate a second wave, since most of the population has not acquired immunity. Even if the epidemic ends in one country, there could still be subsequent waves of infection by imports from abroad unless there is strict quarantine of cross-border travellers. This is the reality of the current COVID-19 pandemic; which approach to take was the difficult decision confronting policy-makers in the early stage of an epidemic. It was reported [8] that the UK first contemplated not suppressing the epidemic through lockdowns, fearing that doing so would only lead to a larger second outbreak because most of the population would not have gained immunity. So, the plan was to let the epidemic run its course while protecting the elderly. But when shown a model prediction [3] that such a ‘do-nothing scenario’ would lead to 500 000 deaths and 81% of the population infected, policy-makers changed course and imposed strict counter measures. This is an important and proper role for a model, to prompt policy actions to combat the spread of the disease. Once the outbreak started, the accuracy of the original model predictions cannot be verified as the forecast forever changed the course of the epidemic in the UK. Health officials in Sweden did not believe in models and decided to pursue ‘herd immunity’ starting 12 March 2020, though this phrase was never mentioned as a policy goal in public statements.

The number of people that will have to be infected before achieving herd immunity depends on how contagious the disease is. The 16 March report of Ferguson *et al*. [3] assumed an infection rate, expressed in terms of basis reproductive number *R*_0_, of 2.4. For the USA, it predicted that 81% of the population would have to be infected for the epidemic to end in this do-nothing scenario, or about 250 million, resulting in 2.2 million dead. Later updates in the 30 March report of Flaxman [9] suggested that *R*_0_ should be about 4 for the European countries studied [10]. Estimating this number for three continents will be one of the tasks in the present work so that one can evaluate what it entails for the herd immunity approach. We will show directly from data that the estimate of *R*_0_ ∼ 4 also holds for the USA, and in fact approximately so for every country we examined, implying an initial e-folding time of 3 days (or doubling time of 2 days). COVID-19 turns out to be much more contagious than originally thought. See also [11].

For South Korea, the epidemic in that country was first suppressed with just 0.02% of its population infected. In Wuhan, the epidemic first ended with less than 0.5% of its population infected. Both are less than 1% of that required to achieve herd immunity as predicted by most models, although it should be pointed out that the above-quoted numbers for Wuhan and South Korea are for the confirmed cases, and do not include the asymptomatic infected. In early April 2020, about 3330 individuals in Santa Clara County in California were tested for antibodies to the COVID-19 virus in their blood [12]. When weighted by demographics and extrapolated statistically to the whole county's population, it was calculated that 2.8% of the population could have been infected. These numbers, less than 5%, are much less than what is required to achieve herd immunity, in a county where the epidemic is waning at the time, probably because of contact reduction measures in place. Similar percentage, 4–5% of the population infected, was found by model up to 4 May 2020 in 11 countries in Europe, when the reproduction number was reduced to below 1 [10]. In early January of 2021, the Chinese Center for Disease Control and Prevention [13] found, through antibody tests, that Wuhan has a prevalence of 4.43%, and China outside Hubei 0.44%. It appears that there was substantial asymtomatic infection in Wuhan. Nevertheless together, it is still one order of magnitude less than that required for herd immunity. It is possible that antibody tests do not reveal all forms of immunity. But the fact that in many countries the emergence of a stronger second and third wave after the first wave has peaked and declined is strong evidence that the decline of the first wave of infections was not a result of herd immunity. Indeed, the situation in these countries represents early examples of the ‘suppressed equilibrium’. Because of its much lower number of deaths, such an end state is a goal that most countries have decided to pursue, despite the enormous toll on the economy due to the much reduced business activity for the two to three months that it would take to achieve it.

Since a ‘suppressed equilibrium’ is achieved in a very different manner from that for the ‘herd immunity’, estimating the end date of the outbreak as a consequence of contact suppression is not based on the number of susceptibles, *S,* approaching a small critical value (i.e. when most of the population is infected, hence acquiring immunity), but on the daily new cases approaching zero and remaining so for two incubation periods, barring new imports. Our estimate of the end of the epidemic is earlier, usually significantly so, because it does not depend on a high percentage of the population having been infected to achieve herd immunity.

In our attempt to reformulate the epidemiological model, we start with more fundamental principles. More fundamental to arguments commonly used to derive SIR-type models are conservation laws. For example, a more fundamental form for the first equation in equation (1.1) should be given by

1.2 $$\frac{\text{d}}{\text{d}t}I=N(t)-R(t),$$

which can be derived rigorously (see §3). It states that the rate of increase of the population of the actively infected is equal to the rate of increase of newly infected, here denoted by *N*(*t*), minus the rate of increase of newly recovered, *R*(*t*), which includes the deaths, together called ‘removed’. In the SIR model, the rate of newly removed does not have a time delay from the rate of newly infected, with both modelled as linearly proportional to *I*(*t*), i.e.

$$\begin{array}{rl} N(t) & \;=aSI(t),\\ R(t) & \;=bI(t)\\ \text{and}\;\;\frac{R(t)}{N(t)} & \;=\frac{b}{aS}>\text{0}. \end{array}\}$$

**Figure 1.**
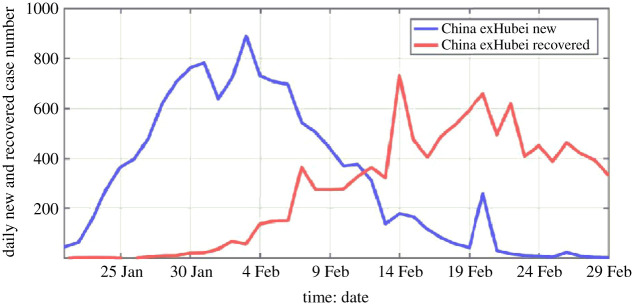
Daily new cases (blue) and daily recovered (red) cases as a function of time for China excluding Hubei. Death is not included in the recovered cases. The horizontal axis is time in date–month. In Hubei, unlike the rest of China, the criterion for confirming COVID-19 infection was changed from the gold standard of nucleic acid test to chest scans on 12 February, in the midst of a rising outbreak, which overwhelmed the test facilities. Data for Hubei and China including Hubei are shown in electronic supplementary material, figure SA2.

This is a statement of instant recovery from infection, which is contrary to observation. Figure 1, from observation, clearly shows a delay of *R*(*t*) relative to *N*(*t*). Here, we use the notation *N*(*t*) to denote the rate of newly infected, which is also called the daily newly infected, although the notation *N* is often used in other models to denote the total population.

**Figure 2.**
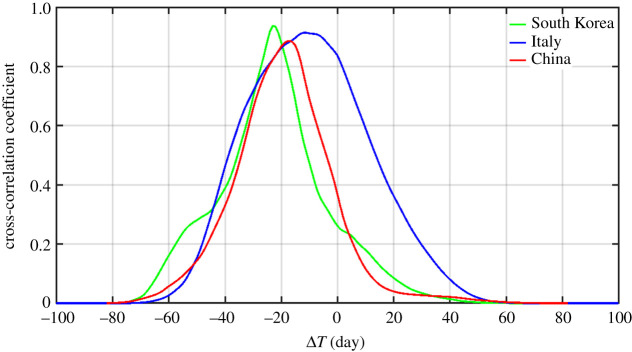
Lagged cross-correlation of *R*(*t*) with *N*(*t*) for China, South Korea and Italy. The maximum correlation of over 0.9 occurs at a lag of *T*. *T* is a statistical mean delay, and varies depending on countries. Deaths are included in *R*(*t*). (Online version in colour.)

Figure 2 shows the cross-correlation of *N*(*t*) and *R*(*t*) for the countries with early outbreaks, China, Korea and Italy. The observed statistics is consistent with a delay of the form

1.3 R(t)=N(t−T),

where *T* is the mean period of recovery, about 10–20 days for the COVID-19 epidemic. Equation (1.3) can be derived rigorously from conservation law (see §3). This conservation law can be easily understood in the case of hospitalizations. A patient admitted to hospital will eventually recover after a mean stay of a couple weeks, or die, after a similar period of time. Also a surge of new patients at some time will likely be followed by a surge of discharges a couple weeks later. We often see such surges in the data.

The model is completed with the mass action law for infection such as: N(t)=aSI(t), as originally proposed by Kermack & McKendrick [1]. Their SIR model, however, should be replaced by equations (1.2) and (1.3). The resulting delay differential equation is much more difficult to solve than the original SIR differential equations without delay, but is much richer in solution behaviour.

It should be pointed out that, unlike the simplified SIR differential equation model (1.1) that later became popular in epidemiology, the original Kermack & McKendrick basic model for the infected population is actually a partial differential equation [1,14] that takes into account the evolution of the infected population through their ‘class age’, i.e. time since first infected. As such, it satisfies partly the von Foerster partial differential equation from age-structured population dynamics that we will use [14–16]. In the partial differential equation version of the model, it is possible to include a time delay of the recovery, but this was not taken into account. In the ordinary differential equation version of the SIR model commonly in use, infected patients are assumed to recover instantaneously. A dependence on the recovery rate on ‘class age’ is not possible in the ordinary equation version of the model. This violation of ‘conservation law’ is not easily noticed because what is calculated in the SIR model is $\tilde{R}$, in the third of equation (1.1), which is the time-integral of *R*. In the current work, we will be solving for the ratio of *N*(*t*) and *R*(*t*). Therefore, their correct relationship is important for our data-driven model.

This paper is organized as follows. We first give in §2 a brief historical review of different types of models. The epidemiological basis for our model is then discussed in §3. The governing epidemiological equations are shown to be equations (1.2) and (1.3). Under the approximation that the change of the susceptible population is not significant, the equations become linear and we find an approximate analytic solution, which is verified to be accurate with numerical calculation in §4. In §5, we discuss various combinations of modelled quantities that can be used to compare with data even though the latter is only for the confirmed cases, while the former includes asymptomatically infected and untested. Based on the properties of the solution, we present a simple prediction tool for epidemic management. It is applied for prediction in §6 and found to be accurate using data for the first waves of the pandemic in several countries. We then return in §7 to some results that are independent of models. There are only two parameters that needed to be specified externally for our model and these are determined from data. The much needed basic reproduction number is also deduced from data for various countries in Asia, Europe and the USA. The theoretical support is developed in the electronic supplementary material, appendix.

## 2. A brief historical review of different types of models

The purpose of our brief review is to put our contribution in the historical context and to relate it to the development over the last few decades of the field of behaviour epidemiology. A more comprehensive review can be found in the 2016 report of Wang *et al*. [17], and the 2013 book edited by Manfredi & d'Onofrio [18].

Each type of models has its strengths and weaknesses. For the mechanistic model, discussed in the Introduction, such as SIR and SEIR or agent-based versions, a key parameter, the infection rate, *a*, is not known for an emerging virus such as SARS-CoV-2, and this has been a source of difficulty with predictions using such models. Some models, such as Imperial College's, treat it as an adjustable parameter to fit the model prediction of the deaths against the data, which is generally thought to be more reliable than that for the infected cases. The infected population is then back-deduced. For the second type of models, especially the purely empirical models without epidemiological basis, it is not known which quantity of the epidemic is predictable.

There have been many empirical models based on the assumption that the progression of daily cases follows a Gaussian ‘epidemic curve’ in time, starting with the early model of William Farr in 1840: ‘Law of Epidemics' in his second annual report to the Registrar General of England and Wales [19]. Lacking the epidemiological mechanisms that Kermack & McKendrick [1] were to later propose, the ‘law’ simply reflected Farr's conviction that the observed deceleration of the rate of increase of infected would not lead to an impending catastrophe but to a crest and then an accelerated decline. The latest is that of the Institute of Health Metrics and Evaluation (IHME) [20]. It turns out that fitting three parameters that define a Gaussian to a short data time series and then using that portion of the Gaussian to predict the subsequent peaks of the epidemic is an ill-posed problem [21]. The uncertainty for prediction several days out was shown to be large [22], unless frequently updated. Later IHME updated to their second generation of model, a traditional SEIR model [23a]. It now uses the death data to back-deduce the infection rate and then runs the model forward for prediction. This inverse problem is discussed in §5a.

The search for the correct ‘geometry of epidemic curves' has a long history in statistical modelling. Farr's Law is purely descriptive, without supplying a mechanism. Farr did not realize that his epidemic curve is Gaussian but nevertheless his descriptive ‘law of second ratios' could be used for prediction, though not accurate. For example (see [23]), if *x*_1_, *x*_2_, *x*_3_, *x*_4_, *x*_5_, *x*_6_… are the successive weekly incidence (i.e. new cases) or mortality, his law says that the ratio of successive ratios of these numbers is a constant, which is less than 1:

$$\frac{X_{4}/X_{3}}{X_{2}/X_{1}}=\frac{X_{5}/X_{4}}{X_{3}/X_{1}}=\cdots =K.$$

That is, there is a constant deceleration of the rate of growth of the cases. After measuring this constant from the early weeks' data, future incidence values could possibly be predicted. It was Brownlee in 1907 [24] who realized that the above formula, when the logarithm is taken—turning the ratio of ratios into difference of differences—is a finite difference form of the second order time-derivative of In *x* being a negative constant [23]:

$$\frac{{\text{d}}^{2}}{\text{d}t^{2}}\text{ln}\;x=\text{ln}\;K<0.$$

Integrating twice and then taking exponentiation would lead to *x*(*t*) being a Gaussian form. Brownlee thought this normal form for the epidemic curve is a fundamental law in epidemiology, but his proposed explanation for the declining growth of the incidence of an epidemic, as due to decreasing ‘infectivity’, was not well received by epidemiologists at the time.

Brownlee [24] provided examples of several epidemics showing that there was fore–aft symmetry in their epidemic curves. For COVID-19, we find that the epidemic curve for Wuhan, China, follows a Gaussian, with near fore–aft symmetry, but that for the USA has a rapid rise but slow decline, definitely not Gaussian. We will show that without human intervention in the form of contact suppression, the epidemiological curve cannot crest and be Gaussian in shape with the low level of infection that exists in most countries. In the modern era of contact suppression, the epidemic curve is shaped by such interventions. We shall explain Wuhan's shape as due to the fact that the contact suppression measures were consistently imposed throughout the course of the outbreak, while in the case of the USA, its states and the populace were relaxing earlier measures on the aft side of the curve, when the new cases declined, creating a fore–aft asymmetry. In the modern era, as countries pursue a ‘suppressed equilibrium’ at great economic cost, there is a tendency in countries with decentralized state governments to relax the countermeasures to various degrees once the disease crested, giving rise to subsequent waves of infection.

Brownlee rejected the idea of herd immunity, that the epidemic's decline was due to ‘an exhaustion of susceptibles', enshrined 20 years later in the SIR model of Kermack & McKendrick [1]. His alternative, ‘infectivity’ idea was based on the thinking that the decline was due to ‘the loss of infecting power on the part of the organism’ [24], and that this biological property of the pathogen (organism) should follow some fundamental law. This biological property of the virus has not been observed in the current COVID-19 pandemic, and does not appear to be a factor. In behavioural epidemiology, Brownlee's definition of ‘infectivity’ is modified to include social factors, since how many people one individual can infect, as measured by the effective reproduction number, *R_t_*, depends on the product of the number of persons contacted during the infectious period and the probability of the contacted person contracting the disease. After implementation of contact reduction measures for a region, we can actually see quantitatively from the data (in §7) the decline of this measure of ‘infectivity’ for that region. The decline is found to be steeper in countries with the more stringent contact-reduction policies and implementation. Although both the mechanisms of loss of susceptibles and decrease in ‘infectivity’ are likely at play, with the extremely small percentage of the population infected in the current pandemic, the second mechanism appears to be the dominant one as countries strive to achieve the ‘suppressed equilibrium’. Given this situation, model predictions of the decline of the epidemic based the fraction of susceptibles decreasing, as in SIR and SEIR models, may be missing the dominant cause for the observed progression of the disease in the current pandemic, although some models empirically adjust the infection rate step-wise in time to reflect changing conditions. Nevertheless, these models may be unnecessarily complex by carrying the nonlinear infrastructure of tracking the susceptible population, which in reality is a very large constant.

Brownlee's idea, with modification expressed above, can be cast in a mathematical form as

2.1 $$x_{t+1}=R_{t}x_{t},$$

where *x_t_* is the incidence (new cases) at time *t*, and *x*_t+1_ is the incidence one infectious period later. An infection period is the duration an infected person remains infectious. *R_t_* is defined earlier as the number of people one infected individual would infect during the period when the individual is infectious. If *R_t_* is a constant, *R_t_* = *R*_0_, the solution to the above finite difference equation is

$$x_{t}=x_{0}{\left(R_{0}\right)}^{t}=x_{0}\exp(t\ln R_{0}),$$

the solution is an unimpeded exponential growth for the relevant case of *R*_0_ > 1. Brownlee [24] commented that such an epidemic form is contrary to the facts: ‘The assumption that the infectivity of an organism is constant, leads to epidemic forms which have no accordance with the actual facts’. With *R_t_* as a decreasing function of time, which we find is actually the case in §7, the above solution becomes Gaussian-like. Specifically, if *R_t_* decreases by a factor *q* < 1 after each period [23,24], due to a ‘loss of infecting power’, i.e. *R_t_* *=* *R*_0_*q*^(*t*-1)^, then the solution is Gaussian

2.2 $$x_{t}=x_{0}{\left(R_{0}\right)}^{t}q^{t(t-1)/2}=x_{0}\exp\left\{t\text{ln}R_{0}+\frac{1}{2}t(t-1)\text{l}\text{n}q\right\},$$

noting ln *q* < 0. Note that in this argument, no mention is made of the decrease of the number of susceptibles; this is not needed when that decrease is small compared to the population as a whole.

Starting from the late 1970s, there has been a rich literature on coupled interaction between humans and disease in the field of behaviour epidemiology, as reviewed by Bauch *et al*. [25]. Recent availability of digital data, such as cellular mobility tracking, has allowed a mapping of social contact networks, leading to models that take into account network topology [17]. Increased computing power then makes such agent-based models feasible.

Our work does not focus on individual behaviour but on country-wide or province-wide responses to policy on contact suppression measures (and the degree of compliance). This effect can be seen readily in the large-scale aggregate data, as we will discuss in §§5 and 7, whereby some countries, such as China and South Korea, have some distinct patterns in their epidemiological curves that can be attributed to the degree of contact suppression, when compared with other regions. We diagnose and attempt to understand these community-wide cause and effects in infectious disease control. By providing a mathematical theory behind these findings, we furthermore provide a validated tool that can be used to monitor and predict the course of the epidemic.

## 3. Our model

We briefly summarize the main ingredients to establish the epidemiological basis of our model. The model is used here to infer general properties of an outbreak, and to discover which properties can be predicted.

All models for epidemics should satisfy certain conservation principles, but many do not. These latter models would be inconsistent with data and, therefore, cannot be used in a data-driven way. One such principle is the delay of the recovered from the infected: a surge in the newly infected should follow some time later, about 20 days for COVID-19, by a similar surge in the newly recovered/removed population (if the deaths are included in the removed category). An auxiliary conservation principle is that the two populations should have equal amplitude in principle. While this is observed in hospitalizations and discharged, those confirmed cases recovering outside the hospital may not be adequately counted and so represent a leakage of the conservation principle in practice. Nevertheless, the conservation principle should be incorporated theoretically in models.

The infected population is governed by the von Foerster partial differential equation in an age-structure population model (see [14–16]). It carries more information than the compartmental models such as SIR or SEIR, but is more difficult to solve.

Let *X*(*t*,*s*) be the number of infected individuals for each ‘age’ at time *t*, with *s* being the ‘class age’, i.e. time since a patient is first infected.

The total number of active infected at time *t* is obtained by integrating over all ages from first infected to recovered/removed:

$$I(t)=\int_{0}^{T}X(t,s)\text{d}s.$$

After being sick for *T* days, a patient either recovers or is removed (dead). *T* is called the recovery period (or removal period). It is also called the infectious period if the patient is infectious during this period. Of course, *T* value varies by patient and by the efficacy of treatment in each hospital. For the removed it also depends on the age of the patient and whether there are underlying medical conditions. Only a mean recovery period is obtainable from data, and so this is in reality a statistical quantity. For a short-duration epidemic, we ignore natural births and deaths. Then the population should be conserved: (d/dt)X(t,s)=0.

This leads to a partial differential equation (see [15]):

3.1 $$\frac{\partial }{\partial t}X+\frac{\partial }{\partial s}X=0.$$

This is the so-called von Foerster equation [14–16] commonly used in population dynamics. It expresses the conservation of the population as it ages, until death. As a population ages, the number of individuals at a certain age *s* changes in time: it decreases as they get older, with their age becoming larger than a particular age *s*, and increases as those who were younger than this age attain this age in time. Here, it is adapted for epidemics by treating *s* as time since infection, the so-called class age, instead of chronological age. For epidemic of short duration, natural birth and death are ignored. The ‘birth’ process is instead the process of first infection and it is modelled as a boundary condition at *s* = 0 (see electronic supplementary material, appendix).

Equation (3.1) can be solved using the method of characteristics to yield

3.2 X(t,s)=G(t−s) for t>s,and 0 for t<s. 

*t* = 0 is the start of the outbreak. The evolution in time of the infected population should vary continuously with the progression of the class age as the infected move through various stages of infection, and such progression should come as a time delay, as expressed by equation (3.2).

Another general principle is equation (1.2)

$$\frac{\text{d}}{\text{d}t}I=N(t)-R(t),$$

where *N*(*t*) is the number of newly infected individuals per day, and *R*(*t*) that of the newly recovered or removed (dead) per day. This equation can be derived from equation (3.1) by integrating it with respect to *s*:

$$\frac{\text{d}}{\text{d}t}I=\int_{0}^{T}\frac{\partial }{\partial t}X(t,s)\text{d}s=-\int_{0}^{T}\frac{\partial }{\partial s}X(t,s)\text{d}s=X(t,0)-X(t,T).$$

We identify *X*(*t*, 0) as the newly infected *N*(*t*), and *X*(*t, T*) as the newly recovered/removed *R*(*t*). The conservation law then follows, since the solution to equation (3.1) is of the form of equation (3.2):

X(t,0)=G(t)for t>0,andX(t,T)=G(t−T)for t>T.

Therefore, for *t* > *T*, the conservation law results: *R*(*t*) = *N*(*t* − *T*).

The distribution of the newly recovery/removal follows that of the newly infected with a time delay of *T*. A more complicated relationship holds for *t* < *T.*

Figure 2, obtained in the early phase of the pandemic using the longest data then available, from China, South Korea and Italy during the COVID-19 pandemic, shows that *N*(*t*) and *R*(*t*) are highly correlated: with correlation coefficients all over 0.9 when both distributions are smoothed with 3-point boxcar. The mean time delay of the correlation can be interpreted as a statistical mean of *T*. The lag time of *R*(*t*) for China is *T* = 19 days, for South Korea is 23 days and for Italy is only 10 days.

Due to their low case fatality rate (CFR), there is practically no difference between the total *R*(*t*) and the recovered for China and Korea. But for Italy, which initially had a high CFR, there are differences between the recovered and the removed. Italy's lag time between *R*(*t*) and *N*(*t*) being shorter than those of China and South Korea does not necessarily mean the shorter the better. The lag time for deaths for China is 7 days, for Korea is 17 and for Italy is only 3 days. This mortality component reduces the overall time for recovery/removal for Italy to 10 days.

With the addition of a ‘constituent law’ for the infection process, such as the ‘mass action law’ of Kermack & Mckindrick [1], *N*(*t*) = *aSI*(*t*), the epidemic model equations are complete:

3.3 $$\frac{\text{d}}{\text{d}t}I=N(t)-N(t-T);\;\frac{\text{d}}{\text{d}t}S=-N(t)\text{.}$$

This is a system of nonlinear ordinary differential equations with delay. For the COVID-19 epidemic, we have argued that the susceptible population has not changed appreciably. So, the last equation is not needed. Absorbing this constant *S* into *a*, and allowing the latter to be a function of time to describe behavioural changes on contact suppression, equation (3.3) becomes

3.4 $$\frac{\text{d}}{\text{d}t}\left(\frac{N(t)}{a(t)}\right)=N(t)-N(t-T).$$

This is a delay differential equation.

A complication could arise if there is a delay between becoming infectious and when an individual is first infected (see electronic supplementary material, appendix). We will not incorporate this additional feature here for the COVID-19 epidemic because of the short latency period of the virus, although this effect can be incorporated without much difficulty.

## 4. Solution

**Figure 3.**
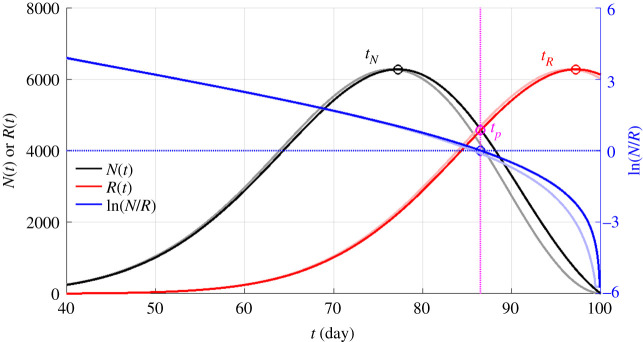
Theoretical epidemic curves: the daily newly infected numbers, *N*(*t*), and the daily recovered numbers, *R*(*t*), are plotted in black and red, respectively. The magnitude is a function of the initial condition, which is arbitrarily chosen here. Also plotted, in blue, is In(*N*(*t*)/*R*(*t*)). It is almost a straight line until near the turning point, where it curves slightly downward. The turning point occurs when this line crosses zero, which is also where *N*(*t*) intersects *R*(*t*). The logarithm becomes negative infinity for *N*(*t*) = 0 for *t* > 100 days after the infection rate becomes zero. To predict the turning point by extrapolating the straight line using the slope from the beginning of the outbreak would overshoot the exact turning point, but only by 2 days, which can still be considered accurate. The darker lines are numerical solutions to equation (3.4) calculated using Mathlab built-in solver dde23 for delay differential equations. The lighter colour lines are the corresponding approximate analytic solution.

Although the delay differential equation is difficult to solve, its numerical solution using canned routines is easy. The analytic solutions, the details of which are relegated to the electronic supplementary material, appendix, help to explain some surprising features of the solution observed in the data, and better reveal the parametric dependence of the solution. We will postpone the discussion on how to determine from data the infection rate until §7. They are calculated here using typical values found from data, with $a(t)=a_{0}-a_{1}t$, $\text{for }0<t<t_{0}$, and a(t)=0, $\text{otherwise}\text{. }a_{0}=0.3\text{ per day}$, $t_{\text{0}}\equiv \frac{a_{0}}{a_{1}}=100\text{ days}\text{.}$ The solution depicted in figure 3 shows that the epidemic curves, *N*(*t*) and *R*(*t*), are Gaussian-like, with the latter being the delayed version of the former, both rising exponentially, cresting at *t_N_* and *t_R_*, respectively, and then decaying. They cross each other at the turning point *t_p_*, which is also the peak of *I*(*t*), when the demand for hospital resources is a maximum. The analytic solutions show that *I*(*t*) is Gaussian, while *N*(*t*) and *R*(*t*) are Rayleigh functions, which are Gaussian-like.

More importantly, the solution has the property that the logarithm of the ratio of *N* and *R* lies on almost a straight line, and that if we extrapolate the straight line from the early weeks of the outbreak, we can predict the turning point with an error of only about 2 days. This result will be verified using data. The analytic solution helps to understand this feature: the ratio of two different Gaussians is another Gaussian unless one Gaussian is a lagged version of the other. Then the quadratic terms in the exponent cancel, and only the linear term remains in exponent. So, the *NR* ratio is an exponential function, whose exponent is a linear function of time. Its logarithm is then a linear function of time.

Some insights gained from the analytic solution are listed below:

ln(*N/R*) lies on a straight line with a zero slope in the absence of contact suppression. This result is derived in electronic supplementary material, SA.7.

**Figure 4.**
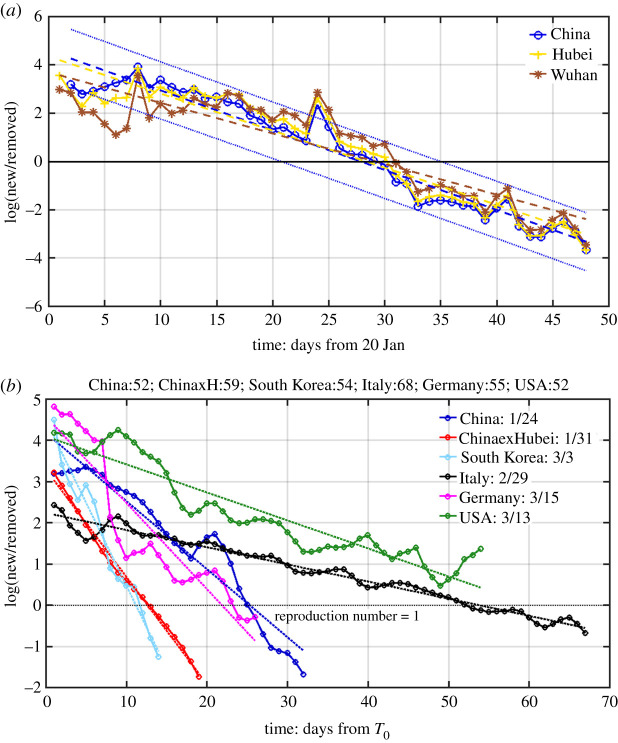
Natural logarithm of the ratio of daily newly infected to newly recovered/removed. They lie on straight lines with some small scatter. The straight line obtained by linear-least-squares fit is in dotted line with the same colour for each country. (*a*) For China, the slopes of the lines are almost the same but with different intercept; the trend lines cross zero (the black horizontal solid line) at different time for different regions indicating different peaking time for AICs. The epicentre Wuhan has later turning point than its province Hubei, which in turn has a later turning point than China as a whole. As a measure of confidence of the linear-least-square fit, the 95% confidence limit for China is given in the figure. Similar confidence limits for other regions have been calculated but are not shown for the sake of clarity of the presentation. (*b*) Comparing different countries. The time is aligned by plotting the *NR* ratio only when the recovery/removal case numbers first exceed 50 (the actual numbers on that day are listed at the top), and *T*_0_ is that calendar date, listed in the inset. Panel (*b*) includes the China cases again, to facilitate comparison with other countries, except the data used were smoothed by a 3-point boxcar filter in (*b*). (Online version in colour.)

For an unchanging infection rate, the solution for *N*(*t*) is exponential growth in time, and since *R*(*t*) *=* *N(t − T)* is the same exponential growth but with time delay, ln(*N/R*) is a constant. This result shows that, without contact suppression intervention, and the observed negative slope of the ln(*N/R*) line found in observation and shown later in figure 4 is not possible, until much later. Of course, the exponential growth will not continue forever; *N*(*t*) will eventually crest and decline due to the depletion of susceptible population.

ln(*N/R*) lies on a straight line with a negative slope in the presence of contact suppression that reduces transmission. See figure 4.

The slope of ln(*N/R*) is $-T/\sigma_{N}^{2}=-a_{1}T,$ where *σ*_N_ is the standard deviation of the Gaussian-like function *N*(*t*). So, $\sigma_{N}=-1/\sqrt{\left(\text{d/d}t)a(t\right)}=1/\sqrt{a_{1}}$ is shown to be inversely proportional to the square root of the negative slope of the infection rate, which measures how strict the contact suppression measures are. These results from our model stand in contrast with those of SIR models.

This result was first discovered empirically from data for countries in Asia and Italy, which had early outbreaks of COVID-19. We later derived it mathematically as solution of the epidemiological equation, supplying the needed epidemiological support. See electronic supplementary material, SA.21, where the following mathematical expression for the slope was derived:

$$\text{ln}\left[\frac{N(t)}{R(t)}\right]≃-\frac{T}{\sigma_{N}^{2}}\cdot t+\frac{1}{2\sigma_{N}^{2}}(T^{2}+2Tt_{p})+\text{ln}\left(1-\frac{T}{t_{0}}\right).$$

The net infection rate, which can be obtained from data, is the same, in the early stage of the outbreak, as the time-varying infection rate that we need for our model.

The former is defined as α(t)=((d/dt)I(t))/I(t), and can be obtained directly from case data.

We show in electronic supplementary material (SA.21) that it is given during the initial period by the infection rate *a*(*t*): $\alpha (t)≃a(t),\text{ for 0 }<t<t_{p}.$

The above result was obtained by ignoring the recovered population and is not valid near the turning point. This however gives a way for us to infer the external parameter *a*(*t*) using data as shown in figure 6 later.

Including the recovered, a complete solution can be found in electronic supplementary material (SA.17), which is

$$\alpha (t)=\frac{a(t)-a(t-T)k(t)}{\left[1-\int_{T}^{t}a(t^{\prime }-T)k(t^{\prime })\text{d}t^{\prime }\right]},$$

where $k(t)\equiv \exp\{-\int_{t-T}^{t}a(t^{\prime })\text{d}t^{\prime }\},a(t-T)=0\text{ for }t<T\text{.}$ In terms of this net infection rate, the solution is

$$I(t)=B\exp\left\{\int_{0}^{t}\alpha (t^{\prime })\text{d}t^{\prime }\right\}\cdot N(t)=a(t)B\exp\left\{\int_{0}^{t}\alpha (t^{\prime })\;\text{d}t^{\prime }\right\}.$$

This result is valid for 0 < *t* < *t_p_* + *T*. This solution is for a general time-dependent infection rate. When restricted to the linear form of *a*(*t*) = *a*_0_ − *a*_1_*t*, it yields a Gaussian for *I*(*t*) and an approximate Rayleigh for *N*(*t*).

## 5. Comparison with observation

### (a) The inverse problem is ill-posed

Most of the model-predicted quantities cannot be compared with observation. A major challenge in modelling the spread of a disease, such as in the COVID-19 epidemic, is that there is a large fraction of undocumented infected population showing no or mild symptoms and, therefore, not likely to be tested and documented as ‘confirmed cases’ [26]. On the other hand, most models predict the total number of infected, whether ‘confirmed’ or not, and this can be orders of magnitude higher than the confirmed cases, at least in Europe [10]. Therefore, it is difficult to compare model predictions with data. Let *p*, the report rate, be the ratio of the confirmed cases to the true infected numbers. If it were known, the case numbers can be divided by *p* to yield the true infected numbers. Because it is largely unknown (i.e. not reported in the databases) and testing policy changes in time, it creates the aforementioned challenge in data-driven models.

In addition to not knowing the report rate *p* to compare model output with the reported case numbers, a key parameter needed in the mechanistic models, the infection rate *a*, is also largely unknown for an emerging disease. There is a large population of asymptomatic, untested and unreported infected individuals, who are nevertheless infectious and produce some of the infected cases reported. The Imperial College group realized this problem early on and their remedy is for their model to predict the deaths and not the infected population [10]. They believe that the death numbers are more reliable. Data on the deaths are still unreliable, as many COVID-19 deaths were attributed to other causes, though they are more reliable than the infection data. The method is to use the death data not only to deduce the death rate, i.e. the rate at which the infected who would die, but also the infection rate, i.e. the rate at which the susceptible population is infected. These are adjusted and the model is rerun until the model simulation for the deaths fits the reported deaths, letting the predicted number of infected be unconstrained by data. The model prediction of the infected population tends to be much higher than the data for the infected cases reported (which is reasonable), as can be seen in their model results. The problem with this approach is that even if the data for the deaths are reliable, this way of fitting data to back deduce the infection rate *a* is too indirect: there may be many factors that affect the death rate other than the infection rate, such as the changing quality of the hospital care, as the healthcare system is being overwhelmed. Mathematically, this is an ill-posed problem, caused by using one set of data (the deaths) to deduce two sets of parameters in the model (the death rate and the infection rate). This difficulty is compounded by the fact that the SIR or SEIR models that they used have instantaneous recovery from infection, which is inconsistent with data.

Fokas *et al*. [27] pointed out that this inverse problem is notoriously difficult, and it is impossible to uniquely identify all parameters of the model given a set of data, even if the available data are reliable. Nevertheless, they showed how a reduced combination of parameters could theoretically be determined given the death time series and all its first four time derivatives, and these could be useful to predict the future deaths, provided that the data time series is long enough, i.e. past the peaks of the epidemic until the accumulated deaths plateau [28]. Their algorithm depends crucially on the reliability and smoothness of the death data, and on the model adopted being correct. Even if the death data are so reliable, the combination of parameters that can be deduced in this inverse problem does not include the parameters that determine the total number of infected.

### (b) Use of ratios in forward instead of inverse approach

In this work, we propose to overcome this problem by using the *NR* ratio, defined as NR(t)=N(t)/R(t).

Let $N{\left(t\right)}_{\text{cases}}=p(t)N(t),$ where *p*(*t*) is the ratio of the confirmed cases to the true infected number. Because of the conservation law, we have

$$NR_{\text{cases}}=\frac{N_{\text{cases}}(t)}{N_{\text{cases}}(t-T)}=\frac{p(t)N(t)}{p(t-T)N(t-T)}=\frac{p(t)N(t)}{p(t-T)R(t)}≃\frac{N(t)}{R(t)}=NR,$$

provided that p(t−T)≃p(t), which is a reasonable assumption, because this ratio does not change in a short period of time, compared with the growth of the infection. Given this reasoning we can compare model-predicted *NR* ratio with the observed *NR* ratio in figure 4.

We show in figure 4, using the case data of the pandemic for COVID-19 for the longest records available at the time of the verification, that the logarithm of *NR*(*t*) ratio lies on a straight line, with small scatter, passing through the turning point *t_p_*. And data for various stages of the epidemic, from the initial exponential growth stage, to near the peak of active infected cases (AIC), and then past the peak, all lie on the same approximate straight line. The intercept with ln(*N*/*R*) yields the turning point. In the real data, *R*(*t*) is affected by treatment efficacy and hospital policies on discharges, and so there is more scatter in the recovered/removed cases. For example, its standard deviation *σ_R_* is slightly larger than *σ_N_*, the standard deviation for *N*(*t*). Here, in the figure, we use the reported recovered/removed cases in the data, and not the theoretical result of *R*(*t*) = *N*(*t* − *T*).

This line, obtained by linear-least-square fit, is little affected by the rather large artificial spike in the data for 12 February for China, because of its short duration and the logarithmic value. That reporting problem is necessarily of short duration because, on the date of definition change, previous week's infected cases according to the new criteria were reported in 1 day. After that, the book is cleared, and *N*(*t*) returned to its normal range.

A comparison of the logarithm of *NR* ratio for several countries is given in figure 4*b*. A steeper slope is associated with an early turning point, and also a predictor for a shorter duration of the epidemic. The shallowest slopes in figure 4*b* were for Italy, where the enormous pressure strained the medical system to the limit, resulting in the largest *σ_R_* value, and one of the highest case fatality rates in the world, at more than 12%. Germany and China have similar slopes. For China outside Hubei, the slope is steepest and the turning point reached 9 days earlier than Wuhan. South Korea's slope is even steeper due to that country's early action. As a result, Italy took a full month longer to reach its turning point than Germany and China, and more than 40 days longer than South Korea.

The observation validates our model result but contrasts the results from SIR model; the latter predicts that the *NR* ratio itself, not its logarithm, should follow a straight line.

Other ratios we use are

$$\alpha (t)=\frac{\text{d}}{\text{d}t}\text{ln}I(t)=\frac{\left(\text{d/d}t)I(t\right)}{I(t)},$$

which crosses zero at *t_p_*, which is the peak of *I*(*t*),

$$\frac{\text{d}}{\text{d}t}\text{ln}N(t)=\frac{\left(\text{d}/\text{d}t)N(t\right)}{N(t)},$$

which crosses zero at *t_N_*, which is the peak of *N*(*t*) and

$$\frac{\text{d}}{\text{d}t}\text{ln}R(t)=\frac{\left(\text{d/d}t)R(t\right)}{R(t)},$$

which crosses zero at *t*_R_, which is the peak of *R*(*t*).

When the report rate *p*(*t*) changes more slowly than the growth of the epidemic, these ratios should be approximately independent of the report rate.

Theoretical justification for these ratios lying on straight lines is given by the fact that *I*(*t*) is a Gaussian. Its logarithm is a quadratic function in time, and therefore becomes linear when differentiated. *N*(*t*) and *R*(*t*) are not exact Gaussians, but Gaussian-like. It can be shown that for these functions, their logarithm is almost a quadratic function and so the derivative of their logarithms lies approximately on a straight line. Verification using case data is given in figure 1*a* in the electronic supplementary material, appendix.

## 6. Predictability

For prediction purposes, instead of curve fitting a Gaussian as some other groups have done, a more accurate and robust prediction tool is based on the ratio of *N*(*t*) and *R*(*t*). This ratio in addition alleviates to some extent the problem related to the data of reported cases being a fraction *p* of the true numbers, as *p* cancels out in the ratio. Unfortunately, some countries, such as the UK and Sweden, do not keep adequate record of *R*(*t*), and many countries do not maintain a rigorous standard, which could be detected by the low case recovery rate, indicating the violation, or leakage, of the conservation law.

Unlike other model predictions, our prediction tool is rather simple and does not require computer simulation. It also does not require that we know the model parameters, such as the infection rate *a*(*t*). Since empirically and theoretically the logarithm of *NR* ratio lies on a straight line passing through the turning point of *I*(*t*), it would be interesting to explore if the turning point, *t_p_*, can be predicted by extrapolation using data weeks before it happened. Extrapolating a straight line is much more practical than other more involved curve fitting algorithms some other groups have adopted. For example, fitting a Gaussian curve was found to have such large uncertainty that the prediction a few days ahead is an ill-posed problem [21]. For our method, how far in advance prediction can be made accurately appears to be limited by the poor quality of the initial data, when *R* is small and highly fluctuating.

**Figure 5.**
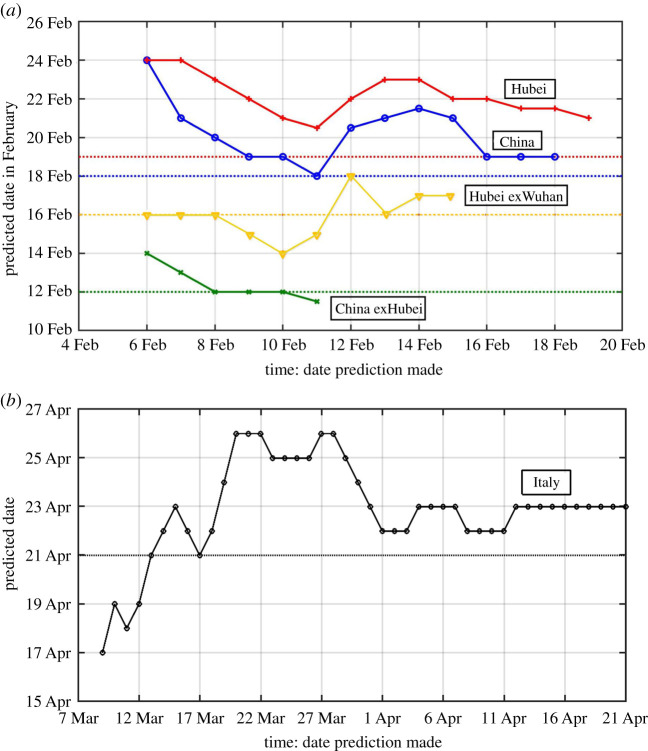
(*a*) Prediction of the turning point in AICs for different regions of China by extrapolating the trends in logarithm of *NR*. The horizontal axis indicates the date the prediction is made using data from 24 January to that date. The vertical axis gives the dates of the predicted turning point. Dashed horizontal lines indicated the true dates for the turning point. (*b*) Prediction of the turning point in AIC for Italy. Data used for all predictions start on 29 February. The first point shown predicts the turning point to occur on 19 April (4 days early) six weeks in advance using 9 days of data from 29 February to 8 March. (Online version in colour.)

Figure 5*a* shows the results of such predictions for China, and figure 5*b* for Italy. It is a hindcast since the truth is now known. The horizontal axis indicates the last date of the data used in the prediction. The beginning date of the data used is 24 January for all experiments for China. Prior to that day, data quality was poor and the newly recovered number was zero in some days, giving an infinite *NR* ratio. For China outside Hubei, the prediction made on 6 February gives the turning point as 14 February, 2 days later than the truth. A prediction made on 8 February already converged to the truth of 12 February, and stays near the truth, differing by no more than fractions of a day with more data.

The huge data glitch on 12 February in Hubei affected the prediction for Hubei, for China as a whole, and for Hubei-exWuhan. These three curves all show a bump up starting on 12 February, as the slope of *N*(*t*) is artificially lifted. Ironically, predictions made earlier than 12 February are actually better. For example, for China as a whole, predictions made on 9 and 10 February both give 19 February as the turning point, only one day off the truth of 18 February. A prediction made on 11 February actually gives the correct turning point that would occur one week later. At the time these predictions are made, the newly infected cases were rising rapidly, by over 2000 each day, and later by over 14 000. It would have been incredulous if one were to announce at that time that the epidemic would turn the corner a week later. Even with the huge spike for the regions affected by Hubei's changing of diagnosis criteria, because of its short duration, the artefact, in which the cases diagnosed by chest X-rays over the previous week were entered as ‘confirmed’ in one day, affects the predicted value by no more than 3 days, and the prediction accuracy soon recovers for China as a whole. For Hubei, the prediction never converges to the true value, but the over-prediction is only 2 days.

For applications to other countries and to future epidemics without a change in the definition of the ‘infection’ to such a large extent, we expect even better prediction accuracy and smaller uncertainty. This better accuracy can be seen in the prediction for Italy. The error of predicting the turning point three weeks in advance is only 1 or 2 days. In fact, a prediction can be made six weeks in advance with an error of 5 days or less.

The prediction for the USA as a whole is less accurate (with errors up to 10 days) because its data are an aggregate of different epicentres. More accurate predictions can be made by treating each state separately. This is not done here because although the data for new cases and deaths are available for each state, recovered data are not individually available. It is also not accurate for the UK because its data for recovered may be suspect.

For countries without an adequate record of *R*(*t*), another, somewhat less robust, method can be used*.* We can obtain *t_N_* and *σ_N_* from data. *t_R_* = *t_N_* + *T*. *T* and *t_p_* cannot be obtained before it occurred, but can be estimated roughly as *t_p_* = *t_N_* + *T*/2, using *T*∼20 days applicable to countries with similar medical systems.

In many countries, pressure mounts for policy-makers to relax the contact-reduction measures when case counts pass the peak and are declining. In some countries where the restrictions are gradually lifted, we should expect a long tail in the epidemic profile. This external influence to the original expected progression of the course should be monitored and adjustment to predictions made in real time. Although we cannot predict policy changes and, therefore, we cannot predict the start of the second wave, once the second wave has progressed for a few weeks, we can make another prediction of the second turning points by starting with a new straight line with a different slope. Second and third waves have now occurred in many countries at this time. However, we have not attempted to make further predictions. What we have shown is a proof of concept that can also be applied to subsequent waves.

Consistent with the above discussion, the relaxation of contact-reduction measures, which changes the slope of the logarithm of *NR* ratio, and lengthens the standard deviation of the new cases, is only significant in the later stages of the course, and can be ignored before the peak, but the prediction on the evolution after the peaks on quantities such as the end of the epidemic and the total number of infected is likely not accurate unless these changes in behaviour are taken into account.

The prediction of the magnitude of AIC at its peak can also be done knowing the predicted turning point and the Gaussian shape of the solution theoretically. See electronic supplementary material, appendix. It is found to be fairly accurate before and near the turning point but not after the turning point, when social distancing and policy may change, and there may be a long tail for newly infected.

## 7. The net infection rate and the reproduction numbers

We present here some results that can be calculated from data independent of models used. We define in general the *net infection rate α*(*t*) as the time-varying exponential growth rate of AIC [29]:

$$\alpha (t)=\frac{\text{d}}{\text{d}t}\text{ln}\;I(t)=\frac{\left(\text{d/d}t)I(t\right)}{I(t)}=\frac{N(t)-R(t)}{I(t)}.$$

This quantity, defined as a ratio of the rate of change of *I* and *I* itself, is not sensitive to the report rate, and therefore, the data for the cases can be used here. The peak number of AIC is a key parameter in the planning for hospital resources. This peak location is called the turning point, denoted by *t_p_*, and can be located in a local-in-time manner using equation (1.2), by when *R* starting to exceed *N*, without first accumulating the data in time to find *I*(*t*). The maximum demand for hospital resources occurs at its peak, and not at the peak of *N*(*t*), although the latter is a more commonly reported quantity.

**Figure 6.**
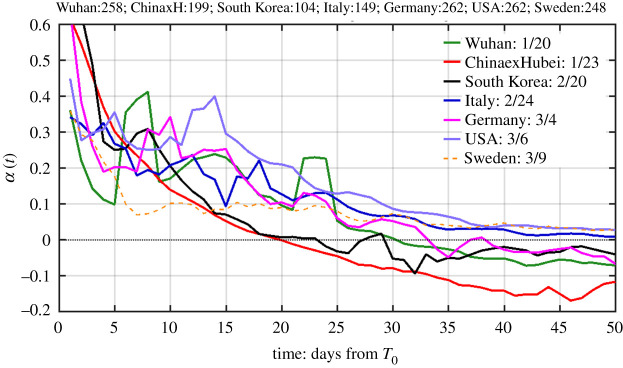
The time-dependent net infection rate (in units of day^−1^) as a function of time starting on the date when the accumulated confirmed case number first exceeds 100 for each region for the first outbreak. The inset legend lists the calendar date (date/month) when each country reaches this milestone. To obtain the actual calendar date, add the dates on the horizontal axis to the starting date indicated in the inset legend. The number of confirmed cases on the starting date is listed at the top. Three day averaging on the raw data has been used. Data source is from Johns Hopkins for all countries, which starts from 23 January 2020, but does not include data for the city of Wuhan, which we obtained from Chinese source. (Online version in colour.)

Figure 6 shows the net infection rate for several countries. The official data that we use include only the confirmed cases (‘cases' for short). In some regions, a subset of cases, those who have more serious symptoms requiring hospitalization, referred to as *total hospitalizations,* are also reported. The peak of total hospitalizations is closely watched by hospital administrators and policy-makers. *α*(*t*) is commonly referred to as *exponential growth rate of active cases* or *hospitalization*. Its inverse gives the e-folding time in days for the cases in an outbreak. A value of say *α* between 0.3 and 0.4 per day, where most countries cluster in the initial period, implies an e-folding time of about 3 days (doubling time of 2 days). The even higher values *α* for many regions in the beginning of our data record may not be due to indigenous disease transmission. See later discussion.

The time for different countries is aligned in figure 6 to begin the time series when each region first exceeded 100 accumulated cases. This way, the progression of the epidemic in each country can be compared. Figure 6 reveals the effects of different policy measures each country adopted. First, South Korea and China exHubei have similar net infection rates (until past their respective turning point); both are much lower than other countries. In the case of South Korea, the government identified early that its epicentre of the epidemic was at church gatherings in the city of Daegu and North Gyeongsang province, where 90% of the initial cases were found. Then aggressive contact tracing was pursued. After the turning point, South Korea soon experienced some second wave episodes, which were successfully contained. These two regions' rigorously implemented contact reduction and aggressive pursuit of ‘Test–Trace–Treat’ measures led to them being the extreme examples of the ‘suppressed equilibrium’.

Germany and Italy have similar exponential growth rates of the net infected case numbers, both slightly higher than Wuhan. More surprisingly, USA has the highest exponential net infection rate, 1.5 times that of Germany and Italy and twice that of Wuhan. This can be attributed to the fact that USA so far does not have a nationwide lockdown, and Europe has had partial lockdowns in phases. Germany took a week longer than Wuhan to reach its turning point. China outside Hubei reached its turning point early, in fact 9 days earlier than the epicentre, Wuhan. This fact is significant, for it is qualitatively different from many traditional model predictions, which had the epicentre achieving its turning point one to two weeks earlier than China outside Hubei [30], probably based on the herd immunity concept.

The net infection rates for China outside Hubei and South Korea are more monotonic than other regions shown. This is likely due to the fact that there was not a piece-meal imposition of social distancing measures, unlike other western countries. The strict measures were imposed and enforced throughout the course. For Wuhan, the large spike on day 23 (12 February) was due to a change in the diagnostic criteria from positive nucleic acid test to chest scan, booking in one day more than 14 000 cases.

To interpret the meaning of the slope of these curves, for countries that had consistent social suppression measures, the net infection rate can be approximated by *α*(*t*) = *α*_0_ – *α*_1_*t*. The negative slope *α*_1_ gives a measure of the effectiveness of the contact suppression in each country. This yields a quadratic exponent and a Gaussian form for *I*(*t*):

$$I(t)=B\exp\left\{\alpha_{0}t-\frac{1}{2}\alpha_{1}t^{2}\right\}=B\exp\left\{-\frac{{\left(t-t_{1}\right)}^{2}-t_{1}^{2}}{2\sigma^{2}}\right\}.$$

The standard deviation of the Gaussian is given by: $\sigma =1/\sqrt{\alpha_{1}}$. The peak is located at $t_{1}=\alpha_{0}/\alpha_{1}$. The slope of the net infection rate determines the width of the Gaussian. Steeper the slope, the narrower is the Gaussian. It also leads to an earlier peak and quicker decline of the epidemic. These results are, however, diagnostic, since *α* is part of the solution. In the electronic supplementary material, appendix, it is solved analytically. We show that the infection rate *a* needed as input for SIR-type models can be inferred from *α* during the early stage of the epidemic, when the recovered population is smaller than the newly infected population. In the electronic supplementary material, appendix, we also solve for *I*(*t*), which has the Gaussian form as diagnosed above.

The case of Sweden needs a special explanation. The epidemic in Sweden initially grows with an e-folding time of around 3 days, in line with other countries. Then on 12 March, the government announced that because of limited resources, it no longer would test for the COVID-19 infection, except for those with serious symptoms already in the hospitals who furthermore were also in the high-risk group. As a result, the new cases took a nose-dive on that day, leading to an artificially low net infection rate of 0.1, implying a 10 day e-folding time. The denominator in the calculation for *α*(*t*) is *I*(*t*), which is an accumulated quantity, and includes those who tested positive prior to 12 March under more liberal criteria. So, this situation explains the flat, low level of *α*(*t*) just above 0. It would eventually cross 0 with large enough death numbers. Sweden's policy decision to implicitly pursue ‘herd immunity’ (while protecting the elderly) has been touted as a viable and perhaps preferable approach to those of other countries in their pursuit of ‘suppressed equilibrium’. It only encouraged those over 70 to stay home and banned visits to nursing homes and gatherings with over 50 people, while business, stores, restaurants and kindergartens through grade 9 were open. The success or failure of this approach cannot be evaluated by the incomplete data. It is noteworthy that, based on the recorded death number, Sweden's *per capita* death toll is 5 and 11 times that of its neighbours Denmark and Norway, respectively.

In traditional mechanistic models, such as the SIR model [1], there is also a time-dependent net infection rate, which at *t* *=* 0, when the population is wholly susceptible, is related to the *basic reproduction number R*_0_. See [31] for a discussion of the complexities associated with this key parameter. We will not be using the SIR model, but it is useful to relate our general definition to what is traditionally used. The equation for *I* in the SIR model is (see equation (1.1))

$$\frac{\text{d}I}{\text{d}t}=aSI-bI=bI\left(\frac{aS}{b}-1\right),$$

where *aS*(*t*) is the infection rate and *b* is the recovery/removal rate.

Therefore

$$\alpha (t)=\frac{\left(\text{d}I/\text{d}t\right)}{I}=b\left(\frac{aS(t)}{b}-1\right)=b(R_{t}-1).$$

For the SIR model, the time-dependent *effective reproduction number* is *R_t_* = *aS*(*t*)/*b*. For the SEIR model, the infected population is (*I*+*E*), where *I* is the infectious and *E* is exposed but not yet infectious. The equation for *I* in SIR model is replaced by

$$\frac{\text{d}}{\text{d}t}(I+E)=bI\left(\frac{aS}{b}-\text{1}\right).$$

The right-hand side remains the same as that for the SIR model. So the reproduction numbers can be defined the same way. Initially, when the whole population is not yet infected, the *basic reproduction number* is $R_{0}=aS(0)/b=R_{t}(0)=\alpha (0)/b+1.$

**Figure 7.**
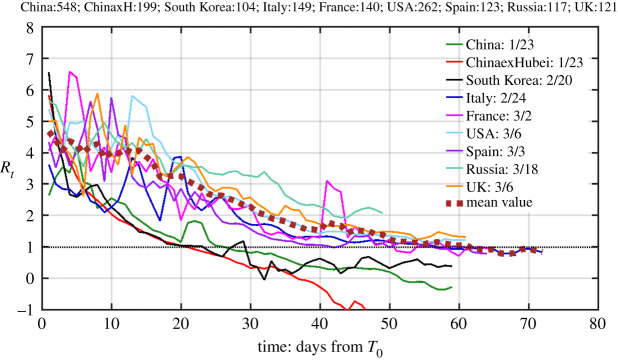
Effective reproduction number for each country or region. The horizontal axis denotes days since day 0 in 2020 (the corresponding calendar date is given in the inset legend), which is the starting date for our calculation. This date is determined by the threshold that the accumulated number of infectives first exceeds 100. The actual number for each region on that day is listed at the top. The thick dashed curve is the average of the curves for the USA and European countries, including Russia. (Online version in colour.)

Our time-dependent net infection rate generalizes this concept to be independent of the SIR or other models: if in the course of an epidemic, *α*(*t*) is positive, the number of infectives will grow exponentially, reaching a peak number of infectives when *α*(*t*) = 0 at *t* = *t_p_*, which is a critical turning point defined previously. Then the total number of active infectives will decrease exponentially. In terms of *R_t_* = *α*(*t*)/*b* + 1, if this number is greater (less) than 1 the total number of active infectives will grow (decrease) at time *t*. We will here use *α*(*t*) directly. *R_t_*, however, is the more watched quantity by the mainstream modellers [31]. It can be calculated from the net infection rate, but will require the determination of an additional parameter *b*, the recovery rate, which may be different for different regions. Furthermore, many countries do not keep adequate records on those who recovered, and so there is an uncertainty in estimating *b.* In figure 7, *R_t_* is obtained by estimating this parameter as $b\approx 1/\sigma_{R}\approx 1/\sigma_{N}$, where *σ_R_* is the standard deviation for the distribution of the daily recovered and *σ_N_* is that for the daily newly infected numbers. *R*_0_ is obtained from *R_t_* in the initial period, before there is significant recovered population.

Figure 7 converts figure 6 to show *R_t_* for each country. Our data-based method shows that *R_t_* clusters around 4 for all countries in three continents during the initial period, consistent with results deduced previously for Europe based on backward simulations of an SEIR model [10]. Because of the problems for the data in the initial period, the curves cannot be extended further back in time to deduce *R*_0_. But based on *R_t_*, a few days later, *R*_0_ for COVID-19 should be around 4, similar to that for SARS. It was originally thought that COVID-19's *R*_0_ was between 2.0 and 2.5 [32], seemingly much less contagious than SARS [33]. Our estimate puts COVID-19 to be much more contagious than the 2009 swine flu pandemic, caused by the H1N1 virus, whose *R*_0_ was estimated [34] to be 1.4–1.6.

In deducing the reproduction numbers, we should not count the large spike for China outside Hubei on day 4. That increase was not due to indigenous transmission, because most of the initial cases were imported from Hubei. This should not be used to infer the reproduction number. Similarly, for South Korea in the first few days shown.

As in figure 6, the decrease of *R_t_* in figure 7 from 4 to 1 for different countries reflects different level of contact-reduction measures adopted and enforced, with China outside Hubei and South Korea sloping more steeply than Europe and the USA. The step-wise behaviour of these numbers for the European countries are rather similar to the model results of Flaxman [9] of Imperial College (their fig. 2), who imposed the parameter changes in steps in their model on the dates they were actually imposed. With their SEIR model calibrated to more accurately predict the mortality, when *R*_0_*∼*4 is used, the modelled death numbers are close to the reported deaths (fig. 2 in [9]).

As we pointed out in the Introduction, the ‘exhaustion of susceptible population’ has not been a factor in the control of the epidemic in many countries, such as China and South Korea, and even in Europe. In models such as SIR and SEIR, the time-dependent decrease of the reproduction number, $R_{t}=aS(t)/b,$ is supposed to be due to the decrease of the susceptible population *S*(*t*). In reality for the COVID-19 epidemic, the decrease should be attributed to human intervention, which results in a decrease in *a*(*t*), with *S*(*t*) deviating not much from the original population for the country.

The UK's record for the recovered is almost non-existent, and what is available shows that the recovered number is only a few per cent of the deaths, which does not appear to be reasonable. Without the recovered in the data, the UK's *R_t_* hovers above 1. There appears to be a similar situation in some other countries, such as Italy. Therefore, the behaviour of *R_t_* in the later stages of the epidemic (in the neighbourhood of the turning point) is probably not correctly depicted by the incomplete data shown for these countries. Nevertheless, in the initial period, when the number of recovered is small, the data shown can be used to estimate *R*_t_ and *R*_0_.

SEIR model was also used to deduce the reproduction number by Institute of Disease Modeling [35] in an effort to monitor the effect of social distancing measures adopted near Seattle (King County, Washington). They found that *R_t_* was reduced from 2.7 to 1.4. Since it was not below 1, the Institute's report advised continuing the measures in place. Since the report rate *p*, that is, the ratio of the number of reported cases versus the true infected number was unknown, the authors assumed a range of values, and obtained $R_{0}∼2.7\pm 0.9.$ One can see from this application of the reproduction numbers how important it is to monitor in real time the progress of policy measures to determine whether it is time to relax the measures in place. And it also shows how difficult it is to infer these numbers, given that the report rate is unknown.

## 8. What is needed to reach herd immunity, with and without vaccines

For a region with an *R*_0_ of 4, what proportion of a wholly susceptible population needs to be vaccinated to achieve herd immunity? The answer is more than 75% if the vaccine is 100% effective against infection of the vaccinated. The 75% threshold is needed to reduce *R_t_* from its initial value of 4 to 1. It is called the ‘critical vaccine coverage’ [17], and can be obtained as *x*_c_ from *R*_0_(1 – *x*_c_) = 1.

If the vaccine is 90% effective in real world situations, this effective rate should be divided into 75% to yield 83%. This includes every man, woman and child. Combined with the vaccine hesitancy in some parts of the population, this is indeed a high threshold to cross.

In the USA, the goal for reopening is when 70% of the adult population is vaccinated. It is seen here that this percentage is not high enough. So social distancing measures should still stay in place.

If the vaccine's effective rate is 75% or lower, herd immunity cannot be achieved with vaccination alone. The final end of the pandemic, if it still happens, may necessarily be the ‘suppressed equilibrium’ through social contact suppression. The vaccine can be useful if applied to the population when the epidemic is near its end, as in China, even if the vaccine effectiveness is lower.

In the absence of vaccines, immunity is alternatively conferred by being infected and recovered. The answer is the same: at least 75% of the population needs to be infected to achieve herd immunity. There have been various estimates based on models for answers to both questions, ranging from 50 to 80%. Our estimate is data driven.

The fact that in many countries *R_t_* was reduced to 1 before the availability of vaccines means that it actually reflects the effect of contact reduction measures in place in those countries. The cresting of the observed daily newly infected *N*(*t*) and then its decline in the first wave of the epidemic in many countries cannot be attributed to the depleted fraction of susceptible population (which was negligible) or to vaccines (which were not yet available).

*An additional remark inserted before publication*: The study in this paper was done during the first wave of the pandemic. The COVID-19 virus has recently mutated to become more contagious. It is now difficult to deduce empirically the basic reproduction number, which is defined for a wholly susceptible population, because some of the population have already been vaccinated or infected with a previous variant of the virus. However, an estimate of the reproduction number can be given. The delta variant is found to be 50% more contagious than the alpha variant, which is in turn 50% more contagious than the original virus that first appeared in Wuhan. Since we found that the original virus has *R*_0_≈4, meaning one person can infect 4, the alpha variant, being 50% more transmissible, would be able to infect 6, giving $R_{0}\approx 6$. The delta variant, being 50% more transmissible than the alpha variant, would have $R_{0}\approx 9$, meaning that one person can infect nine other persons. To reach herd immunity, R*_t_* needs to be reduced from 9 to 1. This requires 99% of the population be vaccinated against this variant with a vaccine that is at least 90% effective. This is an almost impossible task. Therefore, the epidemic, now with the delta variant being the dominant strand, cannot be controlled with vaccines alone. Contact suppression, including masking, needs to be re-imposed, and has been found to be effective in India and the UK in turning the corner against this more contagious variant. Our model, which incorporates the effect of contact suppression, becomes even more relevant.

## 8. Discussion and conclusion

A major advantage of our model, and the tool it provides for predicting turning points and various peaks of the epidemic curve, is its simplicity. Although the mathematical model behind it, being a partial differential equation in time and ‘age’ (days sick since first infected), is more complicated than the ordinary differential equations in compartmental models, such as SIR and SEIR, our results are actually quite simple and easily interpretable. The numerical and analytic solutions we have obtained allow predictions to be made for the course of an epidemic without having to run a computer program. The parameters that our model needs can be determined from data and we have done so for the COVID-19 pandemic in various countries for the first waves of the outbreak. Even these parameters do not need to be specified or deduced for our simple prediction tool, which involves extrapolation along a straight line after the early portion of that line has been established. To solve the dichotomy between data and theory, with the former usually specific to the confirmed cases, and the latter for the total infected, whether they have been tested or not and whether they show symptoms or not, we propose to use the ratio of *N*(*t*) and *R*(*t*) in the dataset for confirmed cases. The ratio between the ‘cases' and the ‘true number of infected’ largely cancels out in the *NR* ratio. The quantity *α*(*t*) for the net infection rate is also obtained as a ratio. The statistics part of our tool is no more than linear-least-squares fit to a straight line, which can be done by medical staff without needing a computer model.

Our model is supported by underlying theoretic foundation and validated by the existing data. Because it is based on general epidemiological principles, we suggest that our approach could be applied not just to the current COVID-19 epidemic, but also generally to future novel epidemics.

Importantly, we made explicit the concept of ‘suppressed equilibrium’ as an end state of an epidemic, in additional to the traditional ‘herd immunity’ state. Based on the traditional mechanistic model, an epidemic wanes after a high percentage of the population is infected and then recovered in the process acquiring immunity. This is the so-called ‘herd immunity’ idea. For COVID-19, which we found to be very contagious, more so than previously thought, the ‘herd immunity’ would require almost all of the population be infected and, therefore, would bring unthinkable toll in the number of people sick and dead. A second way for an epidemic to end is with strict contact suppression measures, so that although a large pool of susceptible population still exists, the portion that an infected person comes in contact with is reduced by the measures adopted, again leading to the effective reproductive number less than 1. Unlike the first state mentioned above, this ‘suppressed’ state is ‘parametrically unstable’ in the sense that if the social distancing measures are relaxed before the epidemic ends or new infection is imported after the first wave ends, the epidemic will rebound, as a large portion of the population is still susceptible. For this second state to be a stable equilibrium, the social distancing measures, and quarantine of cross-border visitors need to be maintained until it is clear that the disease has died off. It is this second state that most countries are now aiming for.

Since it is the goal of most countries to eventually approach the ‘suppressed equilibrium’, it is important to note that the deceleration of the growth of the incidence (daily newly infected) that is observed is not a function of biology, but is a result of contact-reduction, which is social science. The mechanism of the exhaustion of susceptibles is not relevant anymore as the number of infected is such a very small percentage of the susceptible population. Therefore, it is not necessary to be burdened with the nonlinear structure of a model, such as SIR or SEIR, to keep track of the change in susceptibles after the start of the outbreak.

In the USA, a second wave started in May 2020 after the epidemic curve had peaked in April and was declining. This is evidence that the cresting of the epidemic curve and its initial decline were not the result of the recovered population reducing the susceptible population, since if it were so the infection would not have started a new wave. It was caused by the reopening measures that some states started to implement in mid-May.

The prediction we made previously used data up to 5 April 2020 [36]. We could not have predicted in April 2020 that states and some countries were beginning to relax the contact suppression measures, and we did not attempt to do so. The current work used data until early May 2020 [37] and so is only relevant for the first wave of the pandemic. Nevertheless, our method can still be used to monitor the second wave or third wave once it breaks out and we could determine a new slope for the prediction line. This can lead to a prediction of the second or third turning point.

## Data accessibility

All data in this study are publicly available from World Health Organization (WHO) at https://www.who.int/emergencies/diseases/novel-coronavirus-2019/situation-reports/; and on the Daily Brief site of China's National Health Commission at http://en.nhc.gov.cn/. The Korean data are available at https://sa.sogou.com/new-weball/page/sgs/epidemic. Coronavirus COVID-19 Global Cases by Johns Hopkins CSSE at https://gisanddata.maps.arcgis.com/apps/opsdashboard/index.html#/bda7594740fd40299423467b48e9ecf6https://github.com/CSSEGISandData/COVID-19/tree/master/csse_covid_19_data/csse_covid_19_time_series.
